# Navigating Challenges in the Endovascular Treatment of Asymptomatic Aortoiliac Aneurysms: A 10-Year Comparative Analysis

**DOI:** 10.3390/jcm12227000

**Published:** 2023-11-09

**Authors:** Khamin Chinsakchai, Natcha Ketklin, Kiattisak Hongku, Chumpol Wongwanit, Nattawut Puangpunngam, Suteekhanit Hahtapornsawan, Sasima Thongsai, Tossapol Prapassaro, Nuttawut Sermsathanasawadi, Chanean Ruangsetakit, Pramook Mutirangura

**Affiliations:** 1 Division of Vascular Surgery, Department of Surgery, Faculty of Medicine Siriraj Hospital, Mahidol University, Bangkok 10700, Thailand; knatcha32@gmail.com (N.K.); kiattisak.hongku@gmail.com (K.H.); wchumpol@gmail.com (C.W.); sueng058@gmail.com (N.P.); khanitis@gmail.com (S.H.); tossapol.vas@gmail.com (T.P.); nuttawut@gmail.com (N.S.); chaneansi@gmail.com (C.R.); pramook.m@gmail.com (P.M.); 2 Research Department, Faculty of Medicine Siriraj Hospital, Mahidol University, Bangkok 10700, Thailand; sasima.ton@mahidol.edu

**Keywords:** abdominal aortoiliac aneurysm, endovascular treatment, iliac branch device, bell-bottom technique, internal iliac artery embolization, outcomes

## Abstract

Background: Treating an abdominal aortoiliac aneurysm (AAIA) with endovascular methods can be challenging when the internal iliac artery (IIA) is involved. Embolizing the IIA and extending the limb to the external iliac artery (IIAE + EE) to prevent a type 2 endoleak may lead to pelvic ischemic complications. To avoid these complications, strategies that preserve the IIA, such as the bell-bottom technique (BBT) and the iliac branch device (IBD), have been proposed. This study aims to compare the outcomes of these three endovascular approaches for AAIA. Methods: Between January 2010 and December 2019, 174 patients with asymptomatic AAIA were enrolled in this retrospective analysis. They were divided into two groups: 81 patients underwent non-IIAE procedures, and 93 patients underwent IIAE procedures. The iliac limb study group consisted of 106 limbs treated with the BBT, 113 limbs treated with the IIAE + EE, and 32 limbs treated with the IBD. The primary outcomes included the 30-day mortality rate and intraoperative limb complications. The secondary outcomes included postoperative pelvic ischemia, freedom from reintervention, and the overall 10-year survival rate. Results: There was no significant difference in the perioperative mortality rate between the non-IIAE group (0%) and the IIAE group (2.1%), *p* = 0.500. The intraoperative limb complications did not differ significantly between the BBT limbs (7.5%), the IIAE + EE limbs (3.5%), and the IBD limbs (3.1%) groups, *p* = 0.349. The incidence of buttock claudication was significantly greater in the bilateral IIAE + EE group compared to the unilateral IIAE + EE and non-IIAE groups (25%, 11%, and 2.5%, *p*-value < 0.004), and was similar to the incidence of buttock rest pain with skin necrosis (15%, 0%, and 0%, *p* < 0.001). During the 10-year follow-up, the BBT limbs group had a significantly lower rate of iliac limb reintervention free time than the IIAE + EE limbs and the IBD limbs groups (88.7%, 98.2%, and 93.8%, *p* = 0.016). There was no significant difference in the overall 10-year survival rate between the non-IIAE and IIAE groups (51.4% vs. 55.9%, *p* = 0.703). Conclusions: The early and late mortality rates were similar between the non-IIAE and IIAE groups. Preserving the IIA is recommended to avoid pelvic ischemic complications. Considering the higher rate of reintervention in the BBT group, the IBD strategy may be preferred for AAIA.

## 1. Introduction

Endovascular aortic aneurysm repair (EVAR) has become widely accepted as an alternative treatment for abdominal aortic aneurysms (AAA), with a lower rate of early postoperative mortality (1.8% vs. 4.3%, *p* = 0.02) when compared to open repair [1]. Nevertheless, the success rates of EVAR are heavily influenced by the anatomical characteristics of the AAA [2].

In cases where the dilatation of the common iliac artery exceeds 18 mm in males and 15 mm in females, it is considered a common iliac artery aneurysm (CIAA), and occurs concurrently in approximately 10% of all abdominal aortic aneurysms (AAA) [3]. The challenge for EVAR for abdominal aortoiliac aneurysm (AAIA) lies in the involvement of the internal iliac artery (IIA), which requires the embolization of the IIA, and the extension of the graft limbs to the external iliac artery, or strategies for the preservation of the IIA.

Traditionally, IIA embolization with extension of the stent graft to the external iliac artery (IIAE + EE) has been used to prevent retrograde flow from the IIA into the aneurysmal sac, known as a type 2 endoleak, which can lead to a ruptured AAIA. Previous studies have reported serious complications, such as pelvic ischemia, after IIA embolization [4,5,6]. To mitigate these complications, preservation strategies for the IIA, such as the bell-bottom technique (BBT) and the iliac branch device (IBD), have been proposed [7,8]. However, the BBT has limitations, as landing a flared iliac limb graft onto the dilatation of a CIA may result in the secondary dilatation of the distal landing zone over time, leading to a type 1B endoleak [9]. On the other hand, the long-term effects of the BBT have not been well-documented in other research [10]. Furthermore, the precise anatomical requirements for the IBD may limit its usage [11]. While some studies have suggested that the IBD reduces pelvic ischemia, the theoretical benefits of the IBD over the IIAE + EE for preventing colonic ischemia have not been thoroughly studied [12,13].

This study aims to compare the early and late outcomes of these three endovascular approaches for AAIA: internal iliac artery embolization with a stent graft extension to the external iliac artery (IIAE + EE), the bell-bottom technique (BBT), and the iliac branch device (IBD).

## 2. Materials and Methods

This retrospective cohort study utilized data from our institution’s prospective registry of abdominal aortic aneurysms. This study included all patients with asymptomatic AAIA who underwent endovascular treatment. This study was conducted with the approval of our Institutional Review Board (Si 858/2019). Furthermore, this study was conducted in accordance with the STROBE guidelines [14] (Supplementary Table S1).

### 2.1. Patient Selection

Between January 2010 and December 2019, our institute conducted EVAR in a total of 434 asymptomatic patients with AAA. Among these patients, 182 individuals (41.9%) were diagnosed with AAIA, which is defined as an AAA accompanied by a concomitant CIA with a diameter exceeding 20 mm. We collected data on demographics, aneurysm morphology, and operative details, excluding 1 case with a previous aortic surgery, and 7 cases with isolated CIAA. Among the remaining patients, we analyzed 174 patients with a total of 251 iliac limbs in three iliac limb study groups. These groups were based on the surgeon’s preference and patient anatomy, resulting in three different treatment strategies, as follows:

(1) Internal iliac artery embolization with a stent graft extension to the external iliac artery (IIAE + EE): this involved the use of coil embolization to block the hypogastric or internal iliac artery, followed by the deployment of a stent graft to the external iliac artery.
(2) Bell-bottom technique (BBT): this technique used a flared-extension stent graft into the common iliac artery (a flared-extension iliac limb is defined as having a diameter ≥ 24 mm) without IIAE.
(3) Iliac branch device (IBD): inn this approach, one or two iliac branch grafts were placed in the IIA to maintain blood flow.

### 2.2. Operative Techniques

The patients who needed an IIAE + EE underwent a staged bilateral IIAE or a unilateral IIAE with the preservation of the contralateral IIA. Some patients with a bilateral CIAA underwent a bilateral IBD. The IIAE procedures were classified as proximal or distal, based on the placement of the embolization materials. The proximal IIAE occluded the IIA with coil embolization or an Amplatzer vascular plug (Abbott, Plymouth, MN, USA) before the bifurcation, except when an IIA aneurysm or ectasia prevented flush occlusion. In these cases, a distal IIA was performed by interrupting the primary branches feeding the IIA aneurysm sac. For the BBT, the patients diagnosed with AAIA with a concomitant ectasic or aneurysmal CIA with a distal landing zone diameter of 20–25 mm, and at least 20 mm in length, were selected for the procedure. The IBD utilized the Zenith Branch Endovascular Iliac Bifurcation (Cook Medical, Brisbane, QLD, Australia) graft. This graft is extended from a standard EVAR stent graft into the EIA, while also maintaining flow into the ipsilateral IIA through a side branch. The IIA was cannulated from the contralateral femoral artery using a preloaded wire from the side branch. A sheath was advanced over the wire into the reinforced stump of an internal iliac branch. A bridging balloon-expandable covered stent was then inserted into the IIA with a minimum 10 mm landing zone. The successful deployment of the IBD was followed by the placement of a main body endograft in the absence of an adequate proximal landing zone in the CIA, or in the presence of aortic aneurysmal disease.

All the procedures were conducted in either a conventional C-arm-equipped operating room or a hybrid operative theater, depending on the available schedule. Prior to the procedure, antibiotic prophylaxis with Cefazolin (2 g, intravenous) was administered. The patients received either locoregional or general anesthesia. Before deploying the device, an aortoiliac angiogram was performed. The placement of the stent graft followed the instructions for use. At the end of the procedure, an aortoiliac angiogram was performed to confirm the correct position of the endograft, and to ensure that the hypogastric arteries were open for the BBT, IBD, and unilateral IIAE + EE. In the event of technical issues during the procedure, additional maneuvers were performed.

### 2.3. Follow-Up Protocol

After undergoing EVAR for AAIA, all the patients underwent a computed tomography angiogram (CTA) at 1 month, 12 months, and then yearly thereafter, or earlier if necessary (e.g., complications or suspected sac enlargement). Patients with stage III and IV chronic kidney disease (eGFR 30–59 and 15–29 mL/min per 1.73 m^2^, respectively) underwent a standard duplex ultrasound for the evaluation of the aortic stent graft.

### 2.4. Outcomes

The primary outcomes of this study encompass the perioperative mortality between the non-IIAE and IIAE groups, and intraoperative limb complications among the three iliac limb study groups, such as limb occlusion, a type 1B endoleak, and adjunct limb procedures. The secondary outcomes of interest of this study include postoperative complications and outcomes, including pelvic ischemia, characterized by symptoms such as buttock claudication and severe buttock ischemia, freedom of reintervention among the three iliac limb study groups, and the overall survival rate between the non-IIAE and IIAE groups over a 10-year follow-up period. The postoperative complications are reported following established guidelines [15].

Buttock claudication refers to walking-induced buttock pain that causes the patients to stop after a certain distance [5]. Severe buttock ischemia is defined as class 3 in the pelvic ischemia grading system, indicating buttock rest pain with or without skin necrosis and/or colonic ischemia [16].

### 2.5. Statistical Analysis

To ensure that the sample size was sufficient for detecting significant differences among at least two survival curves, a post hoc power analysis was conducted using PASS 2021. The analysis utilized a two-sided log-rank test with 219 limbs, with 113 in the IIAE + EE group and 106 in the BBT group. The test achieved 87.4% power at a 0.05 significance level to identify a hazard ratio of 9.826, when the 5-year proportion of reintervention-free for the IIAE + EE group was 0.991. This time point was chosen as both groups had demonstrated relatively stable reintervention-free rates. Descriptive statistics were employed to express the variables of interest. The categorical variables were reported as numbers and percentages, while the continuous variables were presented as the mean ± standard deviations (SD) for the normally distributed data, or the median (range (min, max)) or interquartile range ((IQR): (Q1, Q3)) for the non-normally distributed data. An independent sample *t*-test was used to compare the means of the continuous variables between the groups, and Pearson’s chi-square test, Yates’ continuity correction, or Fisher’s exact test were used to compare the proportions between the groups for the categorical variables. The Kaplan–Meier method was utilized to calculate the freedom of reintervention and overall survival, and the resulting curves were compared using a log-rank test. The data were recorded and analyzed using IBM SPSS Statistics for Windows (Version 26.0, Armonk, NY, IBM Corp.) released in 2019. A *p*-value below 0.05 was considered statistically significant.

## 3. Results

**Figure 1:**
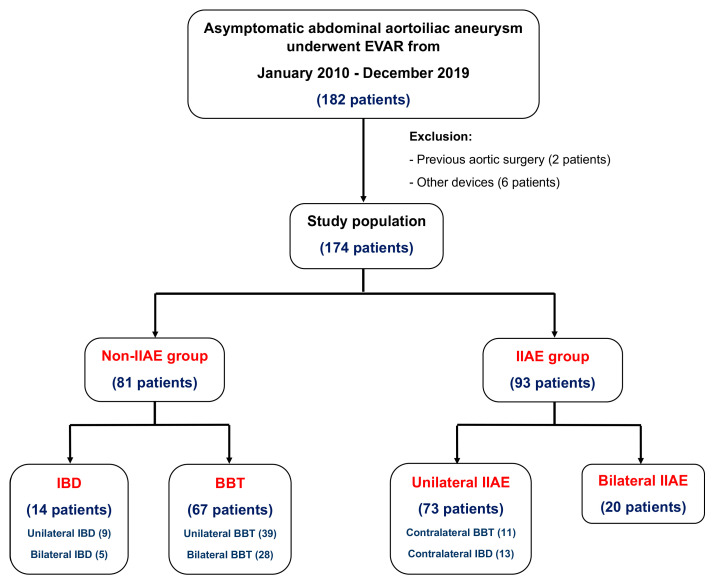
Number and detail of patients between non-IIAE and IIAE groups.

This study included 174 patients with asymptomatic AAIA who had elective endovascular surgery. There was 81 patients in the non-IIAE group and 93 patients in the IIAE group (Figure 1). Non-IIAE was defined as patients who did not undergo embolization on either side of the internal iliac artery, whereas IIAE included patients who underwent embolization either unilaterally or bilaterally in the internal iliac arteries. Most of the patients were male (82.8%), with a median age of 76 years (ranging from 56 to 90 years), and there were no significant differences in either sex or age between the non-IIAE and IIAE groups (76.15 ± 7.23 vs. 74.91 ± 7.48, *p* = 0.272).

The most common comorbidity was hypertension, which did not differ significantly between the two groups. The preoperative hematocrit level, renal function, and serum albumin were similar for both groups. The AAA morphology, including the size, length, neck length, and neck diameter, was also similar between the two groups (Table 1).

**Figure 2:**
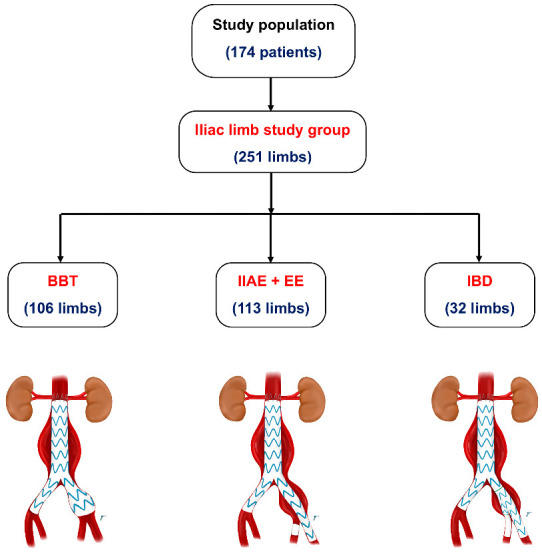
Number of limbs among the 3 endovascular strategy groups.

In this study group of 174 patients, there were a total of 251 common iliac artery aneurysm limbs. These limbs were divided into three groups based on the endovascular treatment strategy: the IIAE + EE limbs (*n* = 113), the BBT limbs (*n* = 106), and the IBD limbs (*n* = 32) (Figure 2). The right side had a slightly higher occurrence of CIA aneurysms compared to the left side (55.8% vs. 44.2%), but this difference was not statistically significant. The mean diameter of the CIA aneurysm in the BBT group was significantly lower than that in the IIAE + EE and IBD groups (23.7 ± 6.3 mm vs. 35.0 ± 13.0 mm vs. 35.5 ± 12.1 mm, *p* < 0.001). Additionally, the IIAE + EE group had a significantly higher percentage of external iliac tortuosity (52.2%) compared to the BBT (29.2%) and IBD (31.3%) groups (*p* < 0.001) (Table 2).

### Operative Details and Outcomes

In the IBD patients, all the devices used were Cook Iliac branch devices (Cook Medical, Brisbane, QLD, Australia). There were no statistical differences in the usage of endografts between the non-IIAE and IIAE groups. When comparing the operative details of the two groups, there was no statistically significant difference in the estimate of blood loss (*p* = 0.089), the fluoroscope time (*p* = 0.732), or contrast usage (*p* = 0.556). Postoperative complications, including organ complications and infection, were comparable between the two groups. The duration of hospitalization did not show significant differences between the non-IIAE and the IIAE groups (*p* = 0.169), as indicated in Table 3. Additionally, the 30-day mortality rate did not show any statistical differences between the non-IIAE and IIAE groups (0% vs. 2.1%, *p* = 0.500).

During the intraoperative period, limb complications were observed. The BBT limbs group had a higher rate of complications at 7.5%, while the IIAE + EE limbs and the IBD limbs groups had rates of 3.5% and 3.1, respectively. However, these differences were not statistically significant (*p* = 0.349). The most common complication was a type 1B endoleak (11/13 = 84.6%). The BBT limbs group required more adjunct limb procedures (7.5%), but this was also not statistically significant. The type 1B endoleak complication was resolved using balloon inflation and the placement of an iliac extension stent. In the IIAE + EE limbs group, one patient experienced an EIA dissection while, in the IBD limbs group, one patient had an EIA occlusion. These cases were addressed using an embolectomy with stent placement in the IIAE + EE limbs group, and a femorofemoral polytetrafluoroethylene bypass graft procedure in the IBD limbs group due to the unsuccessful embolectomy (Table 4).

The median follow-up time was 120 months (IQR, 40.7–120 months). Regarding pelvic ischemic complications (Table 5), buttock claudication was observed in 15 of 174 (8.6%) patients. The bilateral IIAE + EE group had the highest rate of buttock claudication (5/20 = 25%), as compared to the unilateral IIAE + EE (8/71 = 11%) and non-IIAE (2/81 = 2.5%) groups (*p* = 0.004). No cases of severe buttock ischemia were reported in the unilateral IIAE + EE and non-IIAE groups. However, the bilateral IIAE + EE group had a 15% incidence of severe buttock ischemia (*p* < 0.001). Each of these cases experienced buttock rest pain and skin necrosis without colonic ischemia, which resolved within six months through the use of antiplatelet medications and effective wound care. No interruption of the IIA resulted in ischemic colitis requiring laparotomy or causing death.

**Figure 3:**
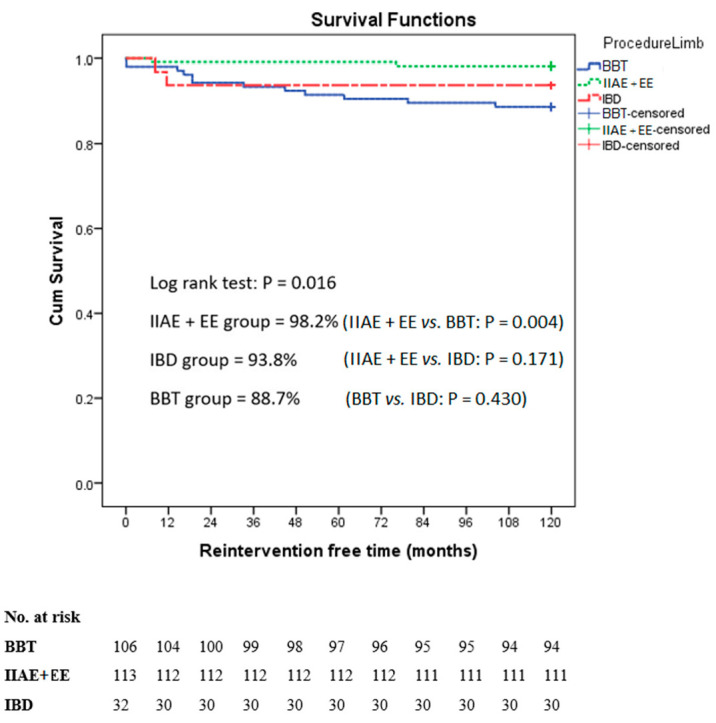
Reintervention-free time among the 3 iliac limb study groups.

**Figure 4:**
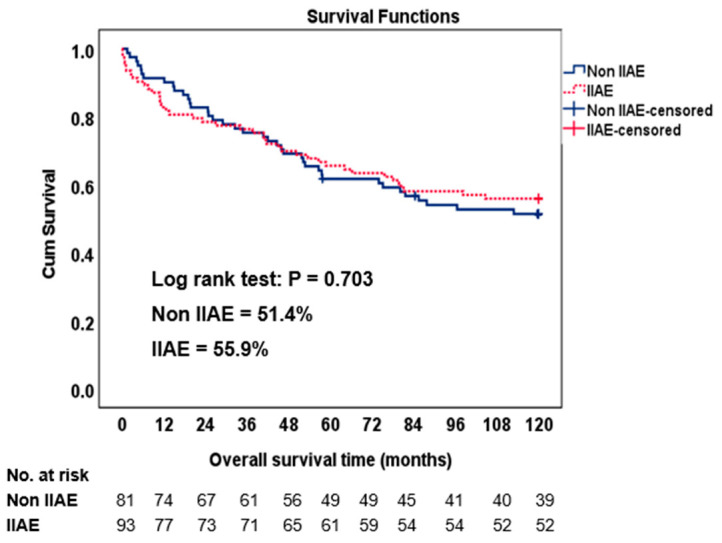
Overall survival between non-IIAE and IIAE groups.

Regarding the limb reintervention-free time after treatment (Figure 3), the log-rank test revealed a significant difference in the reintervention-free time between the three groups (*p* = 0.016). When comparing the combined group of the IIAE + EE (98.2%) with the BBT (88.7%), a significant difference in the reintervention-free survival rate was observed (*p* = 0.004). However, no significant differences were found when comparing the IIAE + EE (98.2%) with the IBD (93.8%), *p* = 0.171, or the BBT (88.7%) with the IBD (93.8%), *p* = 0.430. The most common reason for reintervention in the BBT group after endovascular treatment was a type 1B endoleak (9 out of 12 limbs), which was resolved in 8 limbs with an IIAE+ EE, and in 1 limb with an IBD. Table 6 provides a comprehensive overview of the complications, types of reinterventions, and the timing for each of the three groups. During a 10-year follow-up period (Figure 4), there were no significant differences in the overall survival rates between the non-IIAE (51.4%) and the IIAE groups (55.9%) (*p* = 0.703).

## 4. Discussion

The presence of concurrent CIAA in AAA patients adds complexity to EVAR procedures, compared to cases without CIAA involvement. Our study found that 41.9% of the patients who underwent elective EVAR at our institution had asymptomatic AAA with concurrent CIAA, similar to the 40.2% reported by Bannazadeh et al. [17], but higher than the 16% prevalence reported in the EUROSTAR registry [9]. Our study is the first to compare the outcomes of the three different endovascular approaches for asymptomatic AAIA. Our findings did not show significant differences in the perioperative mortality between the non-IIAE group and the IIAE group. The incidence of intraoperative limb complications did not differ statistically among the BBT limbs, IIAE + EE limbs, and IBD limbs groups. However, the bilateral IIAE + EE group had a significantly higher incidence of buttock claudication and buttock rest pain with skin necrosis, as compared to the unilateral IIAE + EE and non-IIAE groups. During the 10-year follow-up, the overall survival rates did not differ significantly between the non-IIAE and IIAE groups. However, the rate of iliac limb reintervention was significantly higher in the BBT limbs group compared to the IIAE + EE limbs and IBD limbs groups.

Although the baseline characteristics and aneurysm morphology were similar between the non-IIAE group and the IIAE group, the strategies were selected according to surgeon preference and AAIA anatomy. This selection bias must be recognized to enable the correct interpretation of the results. Because the IBD device was not an on-the-shelf device and had to be prepared before use, we excluded the bias of this point by including only the asymptomatic patients who underwent EVAR under elective circumstances. The mean diameter of the CIA aneurysm in the BBT limbs group was smaller than that of the others (about 23 mm and 35 mm); this could be due to the limited maximal diameter of the flare-extension limb of the EVAR stent graft. In the CIA aneurysm with a tortuous external iliac artery, we preferred to choose the IIAE + EE strategy rather than the IBD strategy in order to avoid the potential risk of distal stent IBD graft thrombosis, and also due to the difficulty of the IBD stent deployment. Regarding the postoperative complications, such as the infection rate and organ complication rate, the results of our study were comparable; this could be due to the similarity of the preoperative baseline characteristics and comorbidities between the non-IIAE and IIAE groups.

The lack of a statistically significant difference in the perioperative mortality supports the safety and feasibility of both the non-IIAE and IIAE procedures. Our study did not find any instances of 30-day mortality in the non-IIAE group, which is consistent with previous research on 89 patients who underwent BBT and also reported no 30-day mortality [10]. However, in the IIAE group, there were two patients (2/93 = 2.1%) who did not survive. One patient, who underwent a unilateral IIA embolization, passed away on the seventh day after surgery due to severe sepsis from aspiration pneumonia. The other patient, who had undergone a bilateral IIA embolization, experienced a severe hemorrhage from a retrograde aortic dissection after EVAR, and died on the fifth day after surgery. Our study aligns with previous research on the treatment of asymptomatic AAIA using IIA interruption, which reported a 30-day mortality rate of 1.6% [18]. It is important to note that our study specifically focused on patients with asymptomatic AAIA, enabling a more accurate evaluation of the long-term outcomes. However, further investigations are necessary to determine the most optimal approach for symptomatic AAIA cases, as they may present additional challenges and considerations.

The intraoperative limb complication rates were higher in the BBT limbs group than in the IIAE + EE limbs group and the IBD limbs group (8/106 = 7.5%, 4/113 = 3.5%, and 1/32 = 3.1%, respectively). However, these differences were not statistically significant. The most common complication was a type 1B endoleak in the BBT group treated with balloon inflation and iliac extension stent placement. In general, previous studies have also shown that patients with AAIA had more type 1B endoleaks than those who had AAA without concurrent CIAA [9,17]. Similarly, our study concurs with that of Naughton et al., who compared the results of the BBT vs. IIAE + EE groups and showed that the reintervention and perioperative complication rates were similar (11% vs. 19.1%, *p* = 0.149) between the groups [19].

The preservation strategy for the internal iliac artery, as demonstrated in the non-IIAE group, has shown promising results in reducing the incidence of buttock claudication and severe buttock ischemia. A bilateral IIA embolization resulted in a higher rate of buttock claudication during the follow-up (5/20 = 25%). Unlike a previous study, there were no statistical differences in buttock claudication between the patients with a unilateral IIAE (11%) or a bilateral IIAE (12%) during a 1-year follow-up [20]. Nevertheless, a systematic review confirmed that bilateral IIA occlusion was associated with significantly higher rates of buttock claudication than unilateral interruption (36.5% vs. 27.2%, OR 1.7, 95% CI 1.11–2.6, *p* = 0.01) [8]. All the patients who experienced severe buttock ischemia in this study were also in the bilateral IIAE group (3/20 = 15%). The findings of this study support the recommendation of the preservation strategy, particularly for patients with asymptomatic AAIA.

**Figure 5:**
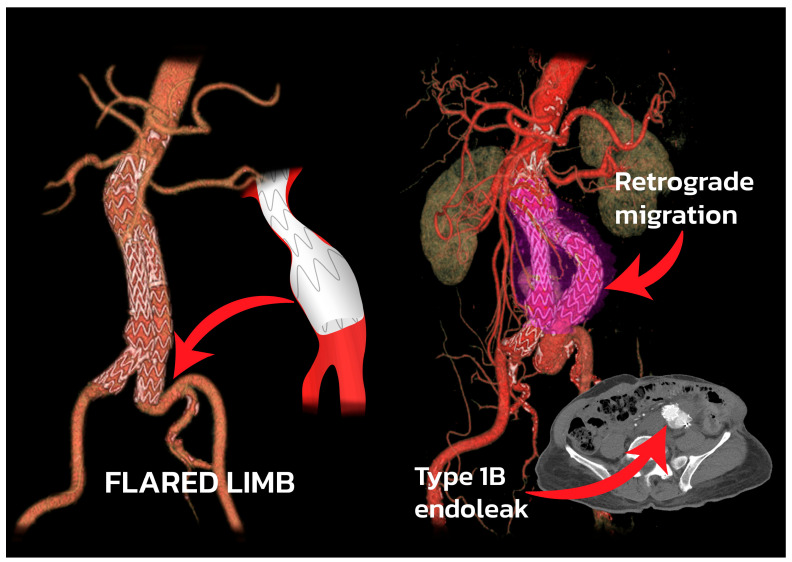
A 73-year-old man was diagnosed with asymptomatic AAIA and underwent EVAR with a 24 mm bell-bottom limb diameter in the left common iliac artery. At the 4-year follow-up, the patient experienced retrograde migration of the left iliac limb graft, leading to a type 1B endoleak.

However, the higher rate of reintervention of the iliac limb observed in the BBT limbs group highlights the importance of considering alternative strategies for the treatment of AAIA. Our findings indicate a significant difference in the reintervention-free time between the groups (*p* = 0.016). The combined group of the IIAE + EE limbs demonstrated a significantly better reintervention-free survival compared to the BBT limbs group (*p* = 0.004), suggesting that the use of IIAE + EE may be associated with improved outcomes in this population. Contrary to Bannazadeh et al. [17], this study showed no significant difference in the reintervention-free device survival between the IIAE + EE, BBT, and iliac limb ≤ 20 mm (95% vs. 91% vs. 93%, *p* = 0.53) over an average follow-up of 59.8 months. However, no significant differences were observed when comparing the group of IIAE + EE limbs with the IBD limbs group (*p* = 0.171) or the group of BBT limbs with the IBD limbs (*p* = 0.430), indicating that the choice between IIAE + EE and IBD may not have a substantial impact on the reintervention-free time. Among the causes of reintervention, a type 1B endoleak was the most common, accounting for 62.5% (10 out of 16 limbs), and the majority occurred in the BBT limbs group. A previous study showed aortic remodeling after EVAR, indicating a connection between a decrease in AAA diameter, an increase in the diameter of the common iliac artery, and aortoiliac elongation resulting in the retrograde displacement of the iliac graft and a type 1B endoleak [21] (Figure 5). Massiere et al. [7] reported a significantly lower freedom from a type 1B endoleak for the BBT group compared to the standard EVAR group. In the IBD limbs group, the reintervention rate was 6.2% (2 limbs) during the ten-year follow-up period. One IBD limb exhibited stent kinking after eight months, resulting in limb stenosis, which was successfully treated with balloon-expandable stent graft relining. Another limb with an IBD experienced a type 3 endoleak, which was corrected using a relining iliac stent graft. The IBD approach, with its branched device design, offers a potential solution for reducing the need for reintervention. The lower rate of reintervention observed in the IBD group suggests that this technique provides better long-term durability and reduces the burden of subsequent procedures for patients. While the IBD strategy necessitates meticulous planning and skill in device deployment, its advantages in terms of minimizing reintervention and pelvic ischemia rates should be taken into account when selecting the optimal treatment approach for AAIA.

The 10-year survival rates did not significantly differ between the non-IIAE and IIAE groups, indicating that both strategies are equally effective in ensuring the long-term survival of patients with asymptomatic AAIA. This finding is reassuring and further supports the viability of the preservation strategy for the internal iliac artery. However, it is important to acknowledge that the overall survival rates observed in this study were relatively modest, emphasizing the need for continued efforts to improve the long-term outcomes for patients with AAIA.

Despite the valuable findings of this study, there are several limitations that need to be acknowledged. Firstly, this was a retrospective study, which may introduce inherent biases and limitations associated with the data collection process. The absence of randomization and the potential for selection bias could impact the generalizability of the results. Secondly, the sample size of the IBD limbs group was relatively small compared to the BBT and IIAE + EE limbs group, which may limit the statistical power and precision of the findings. More studies with larger sample sizes are warranted to validate these results. Lastly, this study focused exclusively on patients with asymptomatic AAIA. Therefore, the findings may not be directly applicable to patients with symptomatic AAIA or those with specific comorbidities. The treatment considerations and outcomes for symptomatic AAIA may differ due to the presence of clinical symptoms and the potential need for urgent intervention. It is important to conduct separate studies to evaluate the efficacy and safety of these endovascular strategies specifically for symptomatic AAIA cases.

## 5. Conclusions

In conclusion, this study provides valuable insights into endovascular treatment strategies for asymptomatic AAIA. The preservation strategy for the internal iliac artery, as demonstrated in the non-IIAE group, offers a viable approach to minimize pelvic ischemic complications and improve postoperative outcomes. The IBD technique, with its lower rate of reintervention, presents a potential alternative for patients who may benefit from enhanced long-term durability. Further research is warranted to validate these findings and to explore the optimal treatment approach for symptomatic AAIA cases. Ultimately, the integration of these strategies into clinical practice has the potential to enhance the management and outcomes of patients with AAIA.

## Figures and Tables

**Table 1:** Patient demographic data, aneurysm morphology, and endografts used in this study.

	Non-IIAE Group	IIAE Group	*p*-Value
	(*n* = 81)	(*n* = 93)	
Age (years) (mean ± SD)	76.15 ± 7.23	74.91 ± 7.48	0.272
Male (*n* (%))	70 (86.4%)	74 (79.6%)	0.233
**Comorbidities (*n* (%))**			
Coronary artery disease	21 (25.9%)	30 (32.2%)	0.360
COPD	10 (12.3%)	13 (13.9%)	0.751
Hypertension	65 (80.2%)	69 (74.3%)	0.344
Dyslipidemia	26 (32.1%)	28 (30.1%)	0.777
Diabetes mellitus	8 (9.9%)	19 (20.4%)	0.055
Cerebrovascular disease	14 (17.3%)	9 (9.7%)	0.139
Chronic kidney disease	10 (12.3%)	14 (15.1%)	0.605
Current smoking	6 (7.4%)	2 (2.1%)	0.612
Hct (%) (median (min, max))	36.9 (24.8, 47.4)	35.1 (21.7, 46.8)	0.326
Creatinine (mg/dL) (median (min, max))	1.28 (0.5, 14.9)	1.18 (0.2, 6.5)	0.091
Albumin (mg/dL) (median (min, max))	3.8 (2.8, 4.9)	3.8 (2.8, 4.7)	0.770
**ASA classification (*n* (%))**			0.758
ASA II	19 (23.4%)	20 (21.5%)	
ASA III	62 (76.5%)	73 (78.5%)	
ASA IV	0 (0%)	0 (0%)	
**Type of aneurysm**			1.000
Fusiform	80 (98.8%)	91 (97.8%)	
Saccular	1 (1.2%)	2 (2.2%)	
**AAA morphology**			
Aneurysm size (mm) (mean ± SD)	61.43 ± 11.46	60.90 ± 13.35	0.779
Aneurysm length (mm) (mean ± SD)	121.36 ± 17.58	119.85 ± 23.64	0.638
Neck diameter (mm) (mean ± SD)	24.59 ± 5.09	24.50 ± 4.28	0.896
Neck length (mm) (mean ± SD)	29.16 ± 14.82	30.7 ± 14.32	0.484
Endografts used *			0.586
Zenith	39 (48.1%)	42 (45.1%)	
Endurant	36 (44.4%)	38 (40.9%)	
Ovation	2 (2.5%)	4 (4.3%)	
AFX	0	2 (2.1%)	
Treovance	4 (4.9%)	7 (7.5%)	

Abbreviations: IIAE: internal iliac artery embolization; *n*: number; SD: standard deviation; COPD: chronic obstructive pulmonary disease; Hct: hematocrit; ASA: American Society of Anesthesiologists; AAA: abdominal aortic aneurysm; mm: millimeter. * Zenith bifurcated stent graft (Cook Medical, Brisbane, QLD, Australia), Endurant abdominal stent graft (Medtronic, Santa Rosa, CA, USA), Treovance abdominal stent graft (Terumo Aortic (formerly Bolton Medical), Sunrise, FL, USA), Ovation abdominal stent graft (TriVascular Inc., Santa Rosa, CA, USA), and AFX (Endoogix Inc., Irvine, CA, USA).

**Table 2:** Iliac artery morphology.

	BBT	IIAE + EE	IBD	*p*-Value
	(*n* = 106)	(*n* = 113)	(*n* = 32)	
**Side of CIAA**				0.452
Right	59 (55.7%)	60 (53.1%)	21 (65.6%)	
Left	47 (44.3%)	53 (46.9%)	11 (34.4%)	
**CIA diameter (mm) (mean ± SD)**	23.7 ± 6.3	35.0 ± 13.0	35.5 ± 12.1	<0.001
**External iliac arteries > 6 mm**	99 (93.4%)	109 (96.5%)	32 (100%)	0.234
**External iliac arteries tortuosity**	31 (29.2%)	59 (52.2%)	10 (31.3%)	0.001
**External iliac arteries calcified**	6 (5.7%)	1 (0.9%)	2 (6.3%)	0.113

Abbreviations: BBT: bell bottom; IIAE + EE: internal iliac artery embolization with extension of the stent graft to the external iliac artery; IBD: iliac branch device; *n*: number; CIAA: common iliac artery aneurysm; SD: standard deviation; mm: millimeter.

**Table 3:** Operative details and perioperative outcomes.

	Non-IIAE Group	IIAE Group	*p*-Value
	(*n* = 81)	(*n* = 93)	
EBL (mL), median (min–max)	200, 30–2000	300, 50–3000	0.089
Fluoroscope time (mins) (mean ± SD)	40.07 ± 30.05	44.56 ± 27.88	0.732
Contrast usage (mL) (mean ± SD)	132.75 ± 90.74	125.91 ± 60.90	0.556
**Organ complications (*n* (%))**	9 (11.1%)	9 (9.7%)	0.757
Renal failure	5	4	
Myocardial infarction	3	3	
Respiratory failure	1	1	
Liver failure	0	1	
**Infective complications (*n* (%))**	11 (13.6%)	18 (19.3%)	0.308
UTI	5	6	
Pneumonia	1	7	
Arthritis	2	1	
Cholecystitis	1	2	
Colitis	0	1	
Pneumonia and UTI	1	0	
Wound infection	1	0	
Septicemia	0	1	
Length of stay (days), median (min–max)	6, 3–120	5, 3–164	0.169
In-hospital mortality (*n* (%))	1 (1.2%)	4 (4.3%)	0.374
30-day mortality (*n* (%))	0	2 (2.1%)	0.500

Abbreviations: IIAE: internal iliac artery embolization; *n*: number; EBL: estimate of blood loss; mL: milliliter; SD: standard deviation; UTI: urinary tract infection; min: minimum; max: maximum; mins: minutes

**Table 4:** Intraoperative limb complications and adjunct procedures.

	BBT	IIAE + EE	IBD	*p*-Value
	(*n* = 106)	(*n* = 113)	(*n* = 32)	
**Intraoperative limb complications**	8 (7.5%)	4 (3.5%)	1 (3.1%)	0.349
Endoleak type 1B	8	3	0	
EIA dissection	0	1	0	
EIA occlusion	0	0	1	
**Adjunct limb procedure**	8 (7.5%)	4 (3.5%)	1 (3.1%)	0.349
Balloon inflation	4	1	0	
Iliac stent extension	4	2	0	
Embolectomy with stent/bypass	0	1	1	

Abbreviations: BBT: bell bottom; IIAE + EE: internal iliac artery embolization with extension of the stent graft to the external iliac artery; IBD: iliac branch device; *n*: number; EIA: external iliac artery.

**Table 5:** Late pelvic ischemic complications among the bilateral IIAE + EE, unilateral IIAE + EE, and non-IIAE groups.

	Bilateral IIAE + EE	Unilateral IIAE + EE	Non-IIAE	*p*-Value
	(*n* = 20)	(*n* = 73)	(*n* = 81)	
**Buttock claudication**	5 (25%)	8 (11%)	2 (2.5%)	0.004
**Severe buttock ischemia**	3 (15%)	0 (0.0%)	0 (0.0%)	<0.001

Abbreviations: IIAE + EE: internal iliac artery embolization with extension of the stent graft to the external iliac artery; *n*: number.

**Table 6:** Early and late limb complications with the types and time to reintervention in the three iliac limb study groups.

Group		Complications	Reinterventions	Time to Reintervention
**BBT (106 limbs)**				
8 limbs		Endoleak type 1B	IIAE + EE	5 days, 14 months, 16 months, 19 months (2), 3 years, 4 years, and 7 years
2 limbs		CIA enlargement	IIAE + EE	5 years and 6 years
1 limb		Endoleak type 1B	IBD	9 years
1 limb		Endoleak type 3	Relining of the iliac stent	4 days
**IIAE + EE (113 limbs)**				
1 limb		Endoleak type 1B from EIA	Relining of the iliac stent	7 months
1 limb		Endoleak type 2 from inadequate IIAE	IIAE + EE	6 years
**IBD (32 limbs)**				
1 limb		Endoleak type 3	Relining of the iliac stent	12 months
1 limb		Stent kinking	Relining of the iliac stent	8 months

Abbreviations: BBT: bell bottom; IIAE + EE: internal iliac artery embolization with extension of the stent graft to the external iliac artery; IBD: iliac branch device; *n*: number; CIA: common iliac artery.

## Data Availability

The data are unavailable due to privacy or ethical restrictions.

## Supplementary Materials

The following supporting information can be downloaded at: https://www.mdpi.com/article/10.3390/jcm12227000/s1, Table S1: STROBE Statement—checklist of items that should be included in reports of observational studies.
