# Supplementary material for: Navigating Challenges in the Endovascular Treatment of Asymptomatic Aortoiliac Aneurysms: A 10-Year Comparative Analysis
Source: J Clin Med. 2023 Nov 9;12(22):7000. doi: 10.3390/jcm12227000 (PMC10672210; doi:10.3390/jcm12227000)
Supplement: Supplementary file 1 [file jcm-12-07000-s001.zip › jcm-2672301-supplementary.pdf]

Table S1. STROBE Statement—checklist of items that should be included in reports of observational studies.

	Item No.	Recommendation	Page No.	Relevant text from manuscript
**Title and abstract**	1	(*a*) Indicate the study's design with a commonly used term in the title or the abstract	1	Methods: Between January 2010 and December 2019, 174 patients with asymptomatic AAIA were enrolled and in this retrospective analysis.
		(*b*) Provide in the abstract an informative and balanced summary of what was done and what was found	2	Methods: Between January 2010 and December 2019, 174 patients with asymptomatic AAIA were enrolled and in this retrospective analysis. They were divided into two groups: 81 patients underwent non-IIAE procedures, and 93 patients underwent IIAE procedures. The iliac limb study group consisted of 106 limbs treated with the BBT, 113 limbs treated with the IIAE + EE, and 33 limbs treated with the IBD. The primary outcomes included the 30-day mortality rate and intra-operative limb complications. The secondary outcomes included postoperative pelvic ischemia, freedom from re-intervention, and the overall 10-year survival rate.<br>Results: There was no significant difference in the perioperative mortality rate between the non-IIAE group (0%) and the IIAE group (2.1%), p = 0.500. The intraoperative limb complications did not differ significantly between the BBT limbs (7.5%), the IIAE + EE limbs (3.5%), and the IBD limbs (3.1%) groups, p = 0.349. The incidence of buttock claudication was significantly greater in the bilateral IIAE + EE group compared to the unilateral IIAE + EE and non-IIAE groups (25%, 11%, and 2.5%, p-value < 0.004), and was similar

				to the incidence of buttock rest pain with skin necrosis (15%, 0%, and 0%, p < 0.001). During the 10- year follow-up, the BBT limbs group had a significantly lower rate of iliac limb reintervention free time than the IIAE + EE limbs and the IBD limbs groups (88.7%, 98.2%, and 93.8%, p = 0.016). There was no significant difference in the overall 10-year survival rate between the non-IIAE and IIAE groups (51.4% vs. 55.9%, p = 0.703).
**Introduction**				
Background/rationale	2	Explain the scientific background and rationale for the investigation being reported	2	The challenge in for EVAR for abdominal aortoiliac aneurysm (AAIA) lies in the involvement of the internal iliac artery (IIA), which requires the embolization of the IIA, and the extension of the graft limbs to the external iliac artery, or strategies for the preservation of the IIA.
Objectives	3	State specific objectives, including any prespecified hypotheses	2	This study aims to compare the early and late outcomes of these three endovascular approaches for AAIA: internal iliac artery embolization with a stent graft extension to the external iliac artery (IIAE + EE), the bell-bottom technique (BBT), and the iliac branch device (IBD).
**Methods**				
Study design	4	Present key elements of study design early in the paper	2	This retrospective cohort study utilized data from our institution’s prospective registry of abdominal aortic aneurysms.
Setting	5	Describe the setting, locations, and relevant dates, including periods of recruitment, exposure, follow-up, and data collection	2	Between January 2010 and December 2019, our institute conducted EVAR in a total of 434 asymptomatic patients with AAA. Among these

				patients, 182 individuals (41.9%) were diagnosed with AAIA, which is defined as an AAA accompanied by a concomitant CIA with a diameter exceeding 20 mm.
Participants	6	(*a*) *Cohort study*—Give the eligibility criteria, and the sources and methods of selection of participants. Describe methods of follow-up<br>*Case-control study*—Give the eligibility criteria, and the sources and methods of case ascertainment and control selection. Give the rationale for the choice of cases and controls<br>*Cross-sectional study*—Give the eligibility criteria, and the sources and methods of selection of participants	2	We collected data on demographics, aneurysm morphology, and operative details, excluding 1 case with a previous aortic surgery, and 7 cases with isolated CIAA. Among the remaining patients, we analyzed 174 patients with a total of 251 iliac limbs in three iliac-limb study groups. These groups were based on the surgeon's preference and patient anatomy, resulting in three different treatment strategies.
		(*b*) *Cohort study*—For matched studies, give matching criteria and number of exposed and unexposed<br>*Case-control study*—For matched studies, give matching criteria and the number of controls per case		
Variables	7	Clearly define all outcomes, exposures, predictors, potential confounders, and effect modifiers. Give diagnostic criteria, if applicable	3	The primary outcomes of this study encompass the perioperative mortality between the non-IIAE and IIAE groups, and intraoperative limb complications among the three iliac-limb study groups, such as limb occlusion, a type 1B endoleak, and adjunct limb procedures. The secondary outcomes of interest of this study include postoperative complications and outcomes, including pelvic ischemia, characterized by symptoms such as buttock claudication and severe buttock ischemia, freedom of reintervention among the three iliac -

				limb study groups, and the overall survival rate between the non-IIAE and IIAE groups over a 10-year follow-up period. The postoperative complications are reported following established guidelines [15].
Data sources/ measurement	8*	For each variable of interest, give sources of data and details of methods of assessment (measurement). Describe comparability of assessment methods if there is more than one group	4	The categorical variables were reported as numbers and percentages, while the continuous variables were presented as the mean ± standard deviations (SD) for the normally distributed data, or the median ([range (min, max))] or [interquartile range: ((IQR): (Q1, Q3))] for the non-normally distributed data. An independent sample t-test was used to compare the means of the continuous variables between the groups, and the Pearson's chi-square test, Yates' continuity correction, or Fisher's exact test were used to compare the proportions between the groups for the categorical variable. The Kaplan–-Meier method was utilized to calculate the freedom of reintervention and overall survival, and the resulting curves were compared using a log-rank test.
Bias	9	Describe any efforts to address potential sources of bias	2	These groups were based on the surgeon's preference and patient anatomy, resulting in three different treatment strategies.
Study size	10	Explain how the study size was arrived at	4	To ensure that the sample size was sufficient for detecting significant differences among at least two survival curves, a post hoc power analysis

	was conducted using PASS 2021. The analysis utilized a two-sided log-rank test with 219 limbs, with 113 in the IIAE + EE group and 106 in the BBT group. The test achieved 87.4% power at a 0.05 significance level to identify a hazard ratio of 9.826 when the 5-year proportion reintervention-free for the IIAE + EE group was 0.991. This time point was chosen as both groups demonstrated relatively stable reintervention-free rates.

Continued on next page

Quantitative variables	11	Explain how quantitative variables were handled in the analyses. If applicable, describe which groupings were chosen and why	4	Descriptive statistics were employed to express the variables of interest. The categorical variables were reported as numbers and percentages, while the continuous variables were presented as the mean ± standard deviations (SD) for the normally distributed data, or the median ([range (min, max))] or [interquartile range: ((IQR): (Q1, Q3))] for the non-normally distributed data. An independent sample t-test was used to compare the means of the continuous variables between the groups, and the Pearson's chi-square test, Yates' continuity correction, or Fisher's exact test were used to compare the proportions between the groups for the categorical variables.
Statistical methods	12	(*a*) Describe all statistical methods, including those used to control for confounding	4	To ensure that the sample size was sufficient for detecting significant differences among at least two survival curves, a post hoc power analysis was conducted using PASS 2021. The analysis utilized a two-sided log-rank test with 219 limbs, with 113 in the IIAE + EE group and 106 in the BBT group. The test achieved 87.4% power at a 0.05 significance level to identify a hazard ratio of 9.826, when the 5-year proportion of reintervention-free for the IIAE + EE group was 0.991. This time point was chosen as both groups had demonstrated relatively stable reintervention-free rates. Descriptive statistics were employed to express the variables of interest. The categorical variables were reported as numbers and percentages, while the continuous variables were presented as the mean ± standard deviations (SD) for the normally distributed data, or the median ([range (min, max))] or [interquartile range: ((IQR): (Q1, Q3))] for the non-normally distributed data. An independent sample t-test was used to compare the means of the continuous variables between the groups, and the Pearson's chi-square test, Yates' continuity correction, or Fisher's exact test were used to compare the proportions between the groups for the categorical variables. The Kaplan–-Meier method was utilized to calculate the freedom of reintervention and overall survival, and the resulting curves were compared using a log-rank test.
		(*b*) Describe any methods used to examine subgroups and interactions	4	The independent sample t-test was used to compare means of continuous variables between groups, and the Pearson chi-square test, Yates' continuity correction, or Fisher's exact test were used to compare proportions between groups for categorical variables.
		(*c*) Explain how missing data were addressed		No missing data in this study
		(*d*) *Cohort study*—If applicable, explain how loss to follow-up was addressed<br>*Case-control study*—If applicable, explain how matching of cases and controls was addressed		No loss follow-up in this study

		Cross-sectional study—If applicable, describe analytical methods taking account of sampling strategy		
		(*e*) Describe any sensitivity analyses	4	A p-value below 0.05 was considered statistically significant.
**Results**				
Participants	13*	(a) Report numbers of individuals at each stage of study—eg numbers potentially eligible, examined for eligibility, confirmed eligible, included in the study, completing follow-up, and analysed	2	Between January 2010 and December 2019, our institute conducted EVAR in a total of 434 asymptomatic patients with AAA. Among these patients, 182 individuals (41.9%) were diagnosed with AAIA, which is defined as an AAA accompanied by a concomitant CIA with a diameter exceeding 20 mm. We collected data on demographics, aneurysm morphology, and operative details, excluding 1 case with a previous aortic surgery and 7 cases with isolated CIAA. Among the remaining patients, we analyzed 174 patients with a total of 251 iliac limbs in three iliac-limb study groups.
		(b) Give reasons for non-participation at each stage	2	We collected data on demographics, aneurysm morphology, and operative details, excluding 1 case with a previous aortic surgery and 7 cases with isolated CIAA.
		(c) Consider use of a flow diagram	5, 7	Figure 1 and 2
Descriptive data	14*	(a) Give characteristics of study participants (eg demographic, clinical, social) and information on exposures and potential confounders	4	Most of the patients were male (82.8%), with a median age of 76 years (ranging from 56 to 90 years), and there were no significant differences in either sex or age between the non-IIAE and IIAE groups (76.15 ± 7.23 vs. 74.91 ± 7.48, p = 0.272).
		(b) Indicate number of participants with missing data for each variable of interest		No missing data
		(c) *Cohort study*—Summarise follow-up time (eg, average and total amount)	9	The median follow-up time was 120 months (IQR,40.7-120 months).
Outcome data	15*	*Cohort study*—Report numbers of outcome events or summary measures over time	2	There was no significant difference in the perioperative mortality rate between the non-IIAE group (0%) and the IIAE group (2.1%), p = 0.500. The intraoperative limb complications did not differ significantly between the BBT limbs (7.5%), the IIAE + EE limbs (3.5%), and the IBD limbs (3.1%) groups, p = 0.349. The incidence of buttock claudication was significantly greater in the bilateral IIAE + EE group compared to the unilateral IIAE + EE and non-IIAE groups (25%, 11%, and 2.5%, p-value < 0.004), and

			was similarly to the incidence of buttock rest pain with skin necrosis (15%, 0%, and 0%, $p < 0.001$). During the 10- year follow-up, the BBT limbs group had a significantly lower rate of iliac limb reintervention free time than the IIAE + EE limbs and the IBD limbs groups (88.7%, 98.2%, and 93.8%, $p = 0.016$). There was no significant difference in the overall 10-year survival rate between the non-IIAE and IIAE groups (51.4% vs. 55.9%, $p = 0.703$).
		Case-control study—Report numbers in each exposure category, or summary measures of exposure	
		Cross-sectional study—Report numbers of outcome events or summary measures	
Main results	16	(*a*) Give unadjusted estimates and, if applicable, confounder-adjusted estimates and their precision (eg, 95% confidence interval). Make clear which confounders were adjusted for and why they were included	No data
		(*b*) Report category boundaries when continuous variables were categorized	7 When comparing the operative details of the between two groups, there was no statistically significant difference in the estimate of blood loss ($p = 0.089$), the fluoroscope time ($p = 0.732$), or contrast usage ($p = 0.556$).
		(*c*) If relevant, consider translating estimates of relative risk into absolute risk for a meaningful time period	No data

Continued on next page

Other analyses	17	Report other analyses done—eg analyses of subgroups and interactions, and sensitivity analyses	9	Regarding the free time limb reintervention-free time after treatment (Figure 3), the log-rank test revealed a significant difference in the reintervention-free time between the three groups (p = 0.016). When comparing the combined group of the IIAE + EE (98.2%) with the BBT (88.7%), a significant difference in the reintervention-free survival rate was observed (p = 0.004). However, no significant differences were found when comparing the IIAE + EE (98.2%) with the IBD (93.8%), p = 0.171, or the BBT (88.7%) with the IBD (93.8%), p = 0.430. The most common reason for reintervention in the BBT group after endovascular treatment was a type 1B endoleak (9

				out of 12 limbs), which was resolved in 8 limbs with an IIAE+ EE, and in 1 limb with an IBD. Table 6 provides a comprehensive overview of the complications, types of reinterventions, and the timing for each of the three groups. During a 10-year follow-up period (Figure 4), there were no significant differences in the overall survival rates between the non-IIAE (51.4%) and the IIAE groups (55.9%) (p = 0.703).
**Discussion**				
Key results	18	Summarise key results with reference to study objectives	11	In the presence of concurrent CIAA in AAA patients, adding complexity to EVAR procedures, compared to cases without CIAA involvement. Our study found that 41.9% of the patients who underwent elective

EVAR at our institution had asymptomatic AAA with concurrent CIAA, similar to the 40.2% reported by Bannazadeh et al. [17], but higher than the 16% prevalence reported in the EUROSTAR registry [9]. Our study is the first to compare the outcomes of the three different endovascular approaches for asymptomatic AAIA. Our findings did not show significant differences in the perioperative mortality between the non-IIAE group and the IIAE group. The incidence of intra-operative limb complications did not differ statistically among the BBT limbs, IIAE + EE limbs, and IBD limbs groups. However, the bilateral IIAE + EE group had a significantly higher incidence of buttock

				claudication and buttock rest pain with skin necrosis, as compared to the unilateral IIAE + EE and non-IIAE groups. During the 10-year follow-up, the overall survival rates did not differ significantly between the non-IIAE and IIAE groups. However, the rate of iliac limb reintervention was significantly higher in the BBT limbs group compared to the IIAE + EE limbs and IBD limbs groups.
Limitations	19	Discuss limitations of the study, taking into account sources of potential bias or imprecision. Discuss both direction and magnitude of any potential bias	13	Despite the valuable findings of this study, there are several limitations that need to be acknowledged. Firstly, this was a retrospective study, which may introduce inherent biases and limitations associated with the data collection process. The absence of randomization and the potential for selection bias could impact the generalizability of the results. Secondly, the sample size of the IBD limbs group was relatively

				small compared to the BBT and IIAE + EE limbs group, which may limit the statistical power and precision of the findings. More studies with larger sample sizes are warranted to validate these results. Lastly, this study focused exclusively on patients with asymptomatic AAIA. Therefore, the findings may not be directly applicable to patients with symptomatic AAIA or those with specific comorbidities. The treatment considerations and outcomes for symptomatic AAIA may differ due to the presence of clinical symptoms and the potential need for urgent intervention. It is important to conduct separate studies to evaluate the efficacy and safety of these endovascular strategies specifically for symptomatic AAIA cases.
Interpretation	20	Give a cautious overall interpretation of results considering objectives, limitations, multiplicity of analyses, results from similar studies, and other relevant evidence	13	This finding is reassuring and further supports the viability of the preservation strategy for the internal iliac artery. However, it is important to acknowledge that the overall survival rates observed in this study were relatively modest, emphasizing the need for continued efforts to improve the long-term outcomes for patients with AAIA.

Generalisability	21	Discuss the generalisability (external validity) of the study results	13	Therefore, the findings may not be directly applicable to patients with symptomatic AAIA or those with specific comorbidities. The treatment considerations and outcomes for symptomatic AAIA may differ due to the presence of clinical symptoms and the potential need for urgent intervention. It is important to conduct separate studies to evaluate the efficacy and safety of these endovascular strategies specifically for symptomatic AAIA cases.
**Other information**				
Funding	22	Give the source of funding and the role of the funders for the present study and, if applicable, for the original study on which the present article is based	14	This research did not receive specific grants from funding agencies in either the public, commercial, or non-profit sectors.

*Give information separately for cases and controls in case-control studies and, if applicable, for exposed and unexposed groups in cohort and cross-sectional studies.

Note: An Explanation and Elaboration article discusses each checklist item and gives methodological background and published examples of transparent reporting. The STROBE checklist is best used in conjunction with this article (freely available on the Web sites of PLoS Medicine at http://www.plosmedicine.org/, Annals of Internal Medicine at http://www.annals.org/, and Epidemiology at http://www.epidem.com/). Information on the STROBE Initiative is available at www.strobe-statement.org.
